# Excitation of the Pre-frontal and Primary Visual Cortex in Response to Transcorneal Electrical Stimulation in Retinal Degeneration Mice

**DOI:** 10.3389/fnins.2020.572299

**Published:** 2020-10-09

**Authors:** Stephen K. Agadagba, Xin Li, Leanne Lai Hang Chan

**Affiliations:** Department of Electrical Engineering, City University of Hong Kong, Kowloon, Hong Kong

**Keywords:** primary visual cortex, pre-frontal cortex, ECoG activity, transcorneal electrical stimulation, retina

## Abstract

Retinal degeneration (rd) is one of the leading causes of blindness in the modern world today. Various strategies including electrical stimulation are being researched for the restoration of partial or complete vision. Previous studies have demonstrated that the effectiveness of electrical stimulation in somatosensory, frontal and visual cortices is dependent on stimulation parameters including stimulation frequency and brain states. The aim of the study is to investigate the effect of applying a prolonged electrical stimulation on the eye of rd mice with various stimulation frequencies, in awake and anesthetized brain states. We recorded spontaneous electrocorticogram (ECoG) neural activity in prefrontal cortex and primary visual cortex in a mouse model of retinitis pigmentosa (RP) after prolonged (5-day) transcorneal electrical stimulation (pTES) at various frequencies (2, 10, and 20 Hz). We evaluated the absolute power and coherence of spontaneous ECoG neural activities in contralateral primary visual cortex (contra Vcx) and contralateral pre-frontal cortex (contra PFx). Under the awake state, the absolute power of theta, alpha and beta oscillations in contra Vcx, at 10 Hz stimulation, was higher than in the sham group. Under the anesthetized state, the absolute power of medium-, high-, and ultra-high gamma oscillations in the contra PFx, at 2 Hz stimulation, was higher than in the sham group. We also observed that the ultra-high gamma band coherence in contra Vcx-contra PFx was higher than in the sham group, with both 10 and 20 Hz stimulation frequencies. Our results showed that pTES modulates rd brain oscillations in a frequency and brain state-dependent manner. These findings suggest that non-invasive electrical stimulation of retina changes patterns of neural oscillations in the brain circuitry. This also provides a starting point for investigating the sustained effect of electrical stimulation of the retina to brain activities.

## Introduction

Electrical stimulation has a long history of clinical applications, ranging from the use of live organisms to deliver electric current to tissues for pain therapy, to the more recent wide usage of implantable devices in clinical settings (Takeda et al., 2017; Capogrosso et al., 2018). Most importantly, brain stimulation paradigms such as transcranial direct current stimulation (tDCS) and repetitive transcranial magnetic stimulation (rTMS) have been severally demonstrated to cause neuromodulatory effects which induce long lasting changes in the brain. Both techniques have been shown to change cortical excitability that persists even after the end of the stimulation period (Wagner et al., 2009; Bailey et al., 2016). However, the resultant effects of these stimulation paradigms are often dependent on the frequency of stimulation which could either increase or decrease cortical excitability (López-Alonso et al., 2014). This is specifically pronounced in rTMS where it has been shown that low frequency rTMS (1 Hz) reduced cortical excitability and at high frequency (5–20 Hz) increased excitability (Gorsler et al., 2003). For tDCS, the resultant effect has mostly been observed with only anodal current stimulation. Research has investigated electroencephalographic (EEG) changes that occur through low intensity anodal and cathodal tDCS in the posterior parietal areas of human subjects (Spitoni et al., 2013). The authors observed only anodal tDCS caused modulation of alpha oscillations that peaked 7.5 min after stimulation in the studied areas. Similarly, another research also used EEG to show that anodal tDCS altered spontaneous activity. The study showed that there was a significant (*p* < 0.01) increase in theta activity in the first few minutes of stimulation. It also reported an increase in alpha and beta oscillations during and following stimulation and a wide spread activation of brain regions was observed following stimulation (Mangia et al., 2014).

The retina is the thin layer of nervous tissue that lines the periphery of the eye. In accordance with its status as an integral part of the central nervous system, the retina is made up of neural circuitry that convert electrical signals of light sensitive cells (photoreceptors) into action potentials that are transmitted to the brain via the optic nerve axons. Despite this important role, the retina is sometimes liable to suffer from degenerative disease such as retinitis pigmentosa (RP). RP is often characterized by rapid structural and functional loss of the outer photoreceptors (Gupta and Huckfeldt, 2017; Verbakel et al., 2018), starting first with the rod photoreceptors and then eventually destroying the cone photoreceptors. The entire degeneration process culminates in total loss of visual perception. Following loss of the photoreceptors, gradual remodeling of the visual pathway occurs, however, it has been reported that the inner retinal neurons remain intact and preserved from destruction (Santos, 1997; Strettoi, 2015). This has shed a light of hope to the scientific research community and to patients affected by rd. Consequently, therapeutic strategies are till date been researched in preclinical and clinical settings with the end goal of restoring partial or complete vision in rd patients. Invariably, there are not many studies that investigate neural activities in the brain after electrical stimulation of the retina. Moreover, in our best knowledge neuromodulatory changes from repetitive prolonged electrical stimulation of the retina has not been investigated in the brain of RP subjects. Specifically, the effect of altering retinal electrical stimulation frequency on cortical activity of RP mice has not been reported. It is known that electrical stimulation paradigms modify neural activity in various ways, either by increasing or decreasing cortical excitability depending on the parameters of stimulation (Winawer and Parvizi, 2016; Mohan et al., 2019). This unique feature to alter cortical activity is being explored for many experimental and therapeutic applications. Again, to our best knowledge, no study has provided information to the behavioral state-dependency of the brain under prolonged (up to 5 days) retinal stimulation.

In the present study we chose the rd10 mouse line because rd10 mice has a homozygous phosphodiesterase 6b mutation (*Pde6b*^rd10/rd10^) which is similar to the mutation found in human RP. Furthermore, unlike rd1 where by the onset of cell death (P8) overlaps with the final differentiation of the retina, rd10 rods begin to degenerate between P18 thus offering a longer therapeutic window (Gargini et al., 2007). Our study used rd10 mice at age P60–P90. From P60 all rods and cones photoreceptors have been reported to be completely degenerated in rd10 (Chang et al., 2002; Gargini et al., 2007). Despite the degeneration of the outer retinal neurons, unique morphological preservation of the inner retinal neurons bipolar and retinal ganglion cells (RGCs) have been demonstrated. Evidence from retinal histology showed protein kinase C positive bipolar cells in rd10 mice at P35, P95, P210, and P360 and it was also discovered that less than 50% of bipolar neurons were destroyed and from P95 onward, the total number of surviving bipolar neurons remained relatively stable (Barhoum et al., 2008; Mazzoni et al., 2008). Similarly, for RGCs their axons and other processes have also been reported to project to brain visual areas long after photoreceptor loss (Lin and Peng, 2013).

The present study addresses the following research question; Will the prolonged electrical stimulation of the retina influence brain activity on a local and global scale, i.e., in the primary visual cortex and non-visual area (pre-frontal cortex), respectively? To this end, we recorded resting or spontaneous electrocorticogram (ECoG) activity arising in neurons of the pre-frontal cortex and primary visual cortex in response to transcorneal electrical stimulation (TES). We analyzed spontaneous ECoG responses of contralateral primary visual cortex (contra Vcx) and contralateral pre-frontal cortex (contra PFx) neurons as a function of varying stimulation frequencies and brain states (awake and anesthetized) in retinal degeneration 10 (rd 10) mice.

## Materials and Methods

### Animal Preparation and Surgery

**FIGURE 1:**
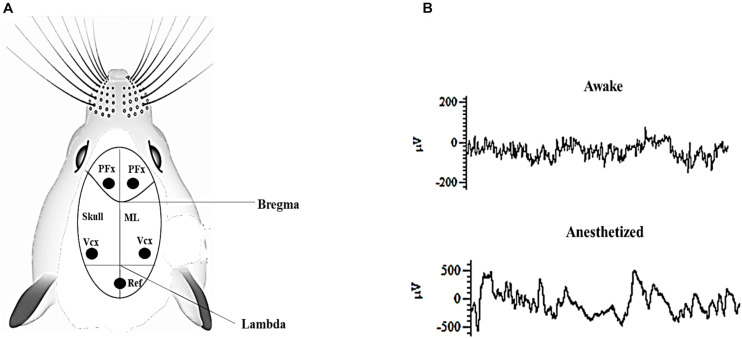
**(A)** Pictorial representation of mouse skull suture lines showing recording sites. PFx, pre-frontal cortex; Vcx, primary visual cortex. Ref: Position of the reference electrode in the cerebellum. ML; Mid-line. **(B)** Representative electrocorticogram (ECoG) raw data trace during awake and anesthetized states after surgery. Both ECoG traces were captured over a period of 1 s.

Mice (rd10) were bred and housed in Laboratory Animal Research Unit of City University of Hong Kong. All procedures were reviewed and approved by the Animal Research Ethics Sub-Committee at City University of Hong Kong and were carried out in compliance with the Animals (Control of Experiments) Ordinance at Department of Health, Hong Kong Special Administrative Region. A total of 40 male and female rd10 mice of post-natal day P60–P90 were used for this study. The surgical procedures were similar to those described in our previous work (Agadagba et al., 2019). Briefly, all animals were initially anesthetized intraperitoneally with ketamine–xylazine mixture (ketamine: 100 mg/kg, xylazine: 10 mg/kg), this was later followed up with administering a combined mixture of isoflurane (1.5%) and medical oxygen (0.4%) in order to maintain the anesthetized state throughout the duration of the surgery. Each animal was stereotaxically implanted (without piercing the dura mater) with stainless steel recording electrodes containing four bone screws and a reference bone screw, each having a shaft diameter of 2.4 mm (Model # 92196A051, McMaster-Carr, NJ, United States). Implantation was done bilaterally over the primary visual cortex (Anterior-Posterior: -3.5 mm, 2.5 mm from the midline) and pre-frontal cortex (Anterior-Posterior: 2 mm, 1 mm from the midline; Figures 1A,B). Throughout the surgery, the heart rate of all animals was monitored and body temperature was kept constant at 37°C with a heating pad (Model # TP702; Gaymar Industries, Inc., NY, United States). Mice eyes were regularly lubricated with lubrithal eye gel to avoid moisture loss during the surgery and recording sessions.

### Impedance Measurement in Phosphate Buffer Saline

In the present study, the impedance of the stimulating electrode (silver wire electrode of diameter: 0.4 mm and surface area: 0.16 mm^2^) was first measured in 0.1 M phosphate buffer saline (PBS) with a constant potentiostat (Model # Reference 600; Gamry Instruments, Warminster, PA, United States) using three-electrode configuration. Briefly, the silver wire electrode was connected to the working electrode input while silver-silver chloride (Ag/AgCl) and platinum (Pt) served as the reference and counter electrodes, respectively. All three electrodes were inserted into 0.1 M PBS and the impedance was detected at an open circuit potential with a 10 mV (root mean square) AC sine wave starting from 1 Hz to 1,000 kHz.

### *In vivo* Impedance Monitoring

*In vivo* electrochemical impedance measurement can be used to sense electrode-tissue proximity and also to estimate whether a constant current is delivered to the tissue during stimulation (Ray et al., 2011). *In vivo* impedance was measured as previously described (Chan et al., 2011). Briefly, the silver wire electrode (diameter: 0.4 mm) was placed on rd10 mice cornea (*n* = 6) and subsequently connected to the working electrode input. Two clip electrodes served as the counter and return electrodes that were positioned on the skin close to the nose and the mouse tail, respectively. The measured impedance was detected with a potentiostat as described above. For the current study, the chosen frequency was 1 kHz. The impedance measurement was monitored for 10 min (at 2 min intervals) before (pre-stimulation impedance) and after (post-stimulation impedance) TES, respectively.

### Transcorneal Electrical Stimulation

Retinal degeneration 10 mice were deeply anesthetized with isoflurane (1.5%) and medical oxygen (0.4%) and were positioned on a stereotaxic apparatus (Stoelting, CA, United States) for electrical stimulation. The silver wire electrode described above (impedance: 0.143 kΩ at 1 kHz) was first connected to an electrical pulse generator (Multi-channel Systems STG stimulator, Model # 4004; Baden-Württemberg, Germany) and then placed transcorneally on the right eye of the mice. The reference needle electrode was placed underneath the skin in close proximity to the stimulated eye. TES was done 30 min per day for a prolonged period of 5 days. The electrical stimuli consisted of charged balanced biphasic square-wave pulses with a pulse duration of 2,000 μs/phase and current intensity of 400 μA. For this study three experimental groups and one sham group was set up. Animals in each experimental group were electrically stimulated with frequencies of 2, 10, and 20 Hz, respectively. The charge injected per phase for each experimental group was 0.8 μC. The sham group was treated in a similar manner as the experimental groups but no current was applied to stimulate the animals.

**FIGURE 2:**
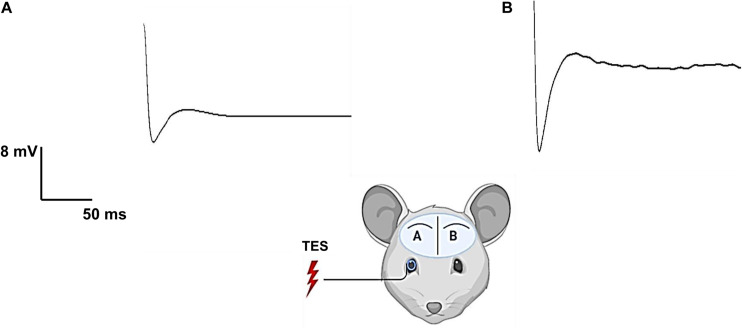
Electrical evoked potential of one rd mouse (rd10) showing activation of retinal-cortical pathway by transcorneal electrical stimulation (TES). The stimulating electrode (silver wire) was placed on the right eye corneal surface and the mouse was stimulated with 400 μA current, 10 Hz frequency and charge balanced biphasic pulse (2 ms/phase). The charge injected per phase was 0.8 μC. Electrical evoked potential was recorded from two locations of the primary visual cortex namely ipsilateral primary visual cortex (Vcx) **(A)** and contralateral Vcx **(B)**. Contralateral Vcx showed more robust response evoked by TES compared to ipsilateral Vcx with lower response.

Previous studies have shown that TES activates the retina circuitry and visual center of the brain, and also demonstrated that the recorded evoked potential in the primary visual cortex originates from the stimulated retina (Timothy and Morley, 2008; Xie et al., 2012). As shown in Figure 2, the electrically evoked ECoG signal was larger in the contralateral visual cortex (Figure 2B) than in the ipsilateral visual cortex (Figure 2A), indicating the visual signal pathway was activated and eliminating the signal transmission through the soft tissues and skull.

### ECoG Recording

**FIGURE 3:**
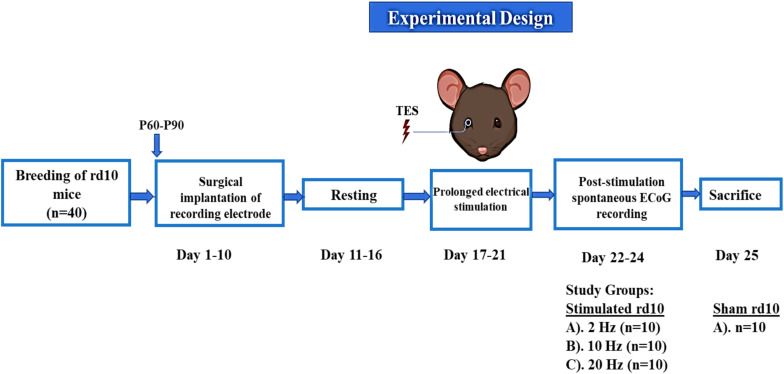
Schematic representation of experimental design for recording of spontaneous ECoG. Each step was done sequentially from breeding of rd10 mice to the end of the experiments in which all animals were sacrificed.

Following TES, resting state or spontaneous activity was recorded over the surface of the brain cortex in the electrode implanted regions using ECoG (Figure 3). The A-M Systems (Model # 3600; A-M Systems, WA, United States) and CED (Model # Micro 1401-3; Cambridge Electronic Design Limited, United Kingdom) were used as the signal amplifier and data acquisition system, respectively. The implanted recording electrode was connected by an active transfer cable to the above set up and ECoG recording was performed under ambient lighting conditions. Post-stimulation spontaneous ECoG signal was filtered from 0.3 to 300 Hz at 5 kHz sampling rate. The recording time was 10 min per day for a total of 3 days in both free moving (awake) and isoflurane-oxygen anesthetized mice. A signal-gain function of 1 K and 50 Hz notch filter was applied in the A-M amplifier during online data acquisition.

### Data Pre-processing and Data Analysis

**FIGURE 4:**
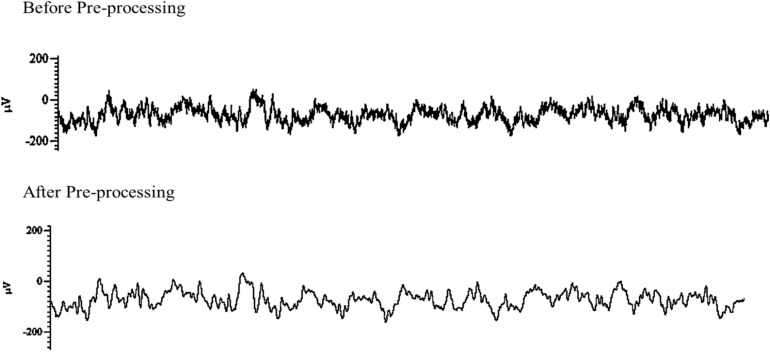
Example data of post-stimulation electroencephalographic (EEG) raw trace before **(top)** and after pre-processing **(bottom)**. Both traces were captured over a period of 5 s.

De-noising and down-sampling were the two main techniques used in this study for data pre-processing (Figure 4). As previously mentioned above, during online sampling 50 Hz notch was applied to remove artifact interfering 50 Hz sinusoidal oscillations and its possible super-harmonics arising from the alternating current line. After data acquisition, a fair amount of noise that observed was removed using MATLAB algorithm (MathWorks, Inc., R2018b, Natick, MA, United States) with the ‘*iircomb*’ function which returns a digital notching filter to remove 50 Hz and its harmonics. ECoG raw data obtained over 10 min recording time generated sufficiently large signals. Each recording electrode channel had approximately 3 × 10^6^ data points, which is very large and increases computation time in MATLAB (MathWorks, Inc., R2018b, Natick, MA, United States). To reduce computation time and increase data processing speed, the data signal was down-sampled from 5 kHz to 500 Hz using MATLAB algorithm (MathWorks, Inc., R2018b, Natick MA, United States).

### Analysis of Absolute Power

Absolute power was estimated from the power spectrum calculated based on discrete Fourier transform with 2-s segment size and 1 s overlapping for individual frequency bin (0.5–300 Hz with 0.5 Hz bin size; ‘spectrogram.m’ function in MATLAB signal processing toolbox; MathWorks, Inc., R2018b, Natick, MA, United States). Hamming window was applied for each segment. The mean and standard error of the mean (SEM) of absolute power was calculated for eight frequency bands namely: delta (0.5–5 Hz), theta (5–10 Hz), alpha (10–15 Hz), beta (15–25 Hz), low gamma (gamma 1; 25–55 Hz), medium gamma (gamma 2; 80–115 Hz), high gamma (gamma 3; 125–145 Hz), and ultra-high gamma (gamma 4; 165–300 Hz).

### Analysis of Coherence

The coherence between the contra Vcx and contra PFx ECoG channels was calculated by magnitude squared coherence *Cxy(f; ‘mscohere.m’* function) in MATLAB signal processing toolbox (MathWorks, Inc., R2018b, Natick, MA, United States). This gives an estimated coherence value of the input signal x and y using the method of Welch’s average modified periodogram. It is important to note that *Cxy(f)* acts as a function of frequency and has values ranging from 0 to 1 which is an indication of the extent to which *x* correlates with *y* per frequency.

(1) $$C\,x\,y\,\left(f\right)=\frac{{\left[P\,x\,y\,\left(f\right)\right]}^{2}}{P\,x\,x\,\left(f\right)\,P\,y\,y\,\left(f\right)},0\leq C\,x\,y\,\left(f\right)\leq 1$$

In Eq. (1) above, *Pxx(f)* and *Pyy(f)* represent the power spectral density of *x* and *y* and *Pxy(f)* represents the cross-power spectrum spectral density. For each mouse, the mean coherence between the contra Vcx and contra PFx was calculated. ECoG signal was split into 2-s epochs with 1-s overlapping over the entire ECoG recording. Subsequently, *Cxy(f)* was estimated at each epoch and frequency bin over the entire 0.5–300 Hz frequency range for each mouse. The mean coherence between contra Vcx and contra PFx was computed for all mice in both awake and anesthetized states.

### Statistical Analysis

Subsequent to data analysis, the data was tested for normal distribution by Shapiro–Wilk test. All graphs were plotted and statistical analysis was done using Origin(Pro) (Version 2015, OriginLab Corporation, Northampton, MA, United States). For *in vivo* impedance monitoring, paired students’ *t*-test was used to test the significance between pre-stimulation and post-stimulation impedance in rd10 mice. *P*-values < 0.05 were indicated as significant. For ECoG recording experiments, test for significance between stimulation groups and sham group was done and *p*-values were calculated using the non-parametric Mann–Whitney *U*-test. Subsequently *p*-values were adjusted by Holm–Bonferroni and significant *p*-values were denoted with asterisks (^∗^*p* < 0.025).

## Results

### Impedance Monitoring

**FIGURE 5:**
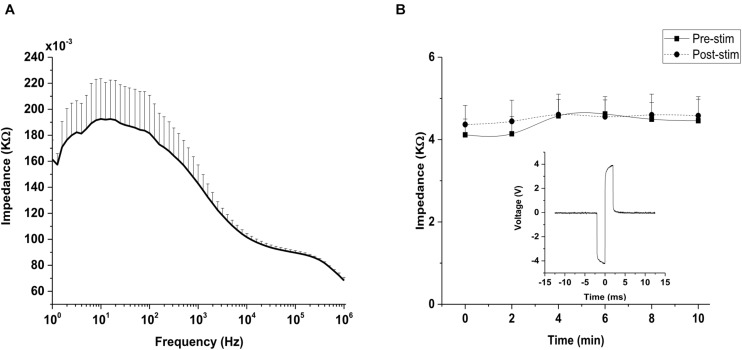
Impedance monitoring. **(A)** Impedance measurements of silver wire electrode in 0.1 M phosphate buffer saline (PBS). Impedance magnitude was measured across a wide range of frequencies (1 Hz–1,000 kHz). Frequency is represented on a log scale (log_10_). **(B)** Representative plot of impedance at the interface between silver wire electrode and rd10 mice cornea (*n* = 6) measured at 1 kHz. No significant difference (*p* > 0.05) was observed between the pre-stimulation and post-stimulation impedance monitored *in vivo* at 1 kHz. Inset figure: Voltage waveform recordings of a silver wire electrode to 400 μA biphasic stimulus pulses recorded at the rd10 mice cornea (measurement was captured from an oscilloscope).

Impedance value for the silver wire electrode was (0.143 ± 0.014) kΩ at 1 kHz, as shown in the impedance spectrogram (Figure 5A). For *in vivo* impedance monitoring, impedance traces showed a constant impedance with no significant difference (*p* = 0.078) between the impedance during pre-stimulation and post-stimulation stages of TES (Figure 5B), indicating that a relatively stable voltage level was applied to the eye. Voltage waveform recordings to 400 μA biphasic stimulus pulses recorded at the electrode-cornea interface was 8 volt (peak-peak; Figure 5B inset).

### Effects of Repetitive Prolonged TES on Spontaneous Absolute Power in Rd10 Mice

**FIGURE 6:**
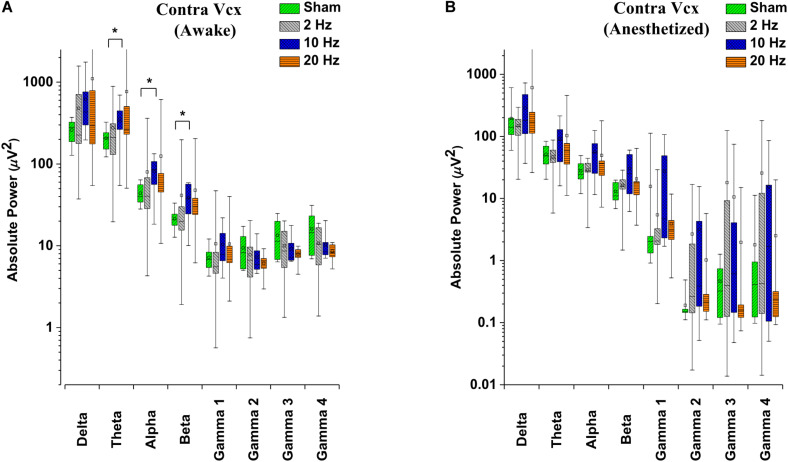
Box-plots showing post-stimulation changes in spontaneous absolute power in the primary visual cortex of rd10 mice during awake **(A)** and anesthetized **(B)** brain states. Absolute power is represented in a log scale. All results were compared to the sham group. All *p*-values were adjusted by Holm–Bonferroni method. * denotes significant change in spontaneous absolute power compared to sham group. Short horizontal lines embedded in each boxplot represent the median while the small square symbols represent the mean spontaneous absolute power of each group. Sham (*n* = 8), 2 Hz (*n* = 8), 10 Hz (*n* = 8), 20 Hz (*n* = 9).

Alterations in spontaneous absolute power following 5 days of repetitive prolonged TES in awake and anesthetized rd10 mice was investigated using ECoG. In contra Vcx of awake rd10 mice, 10 Hz stimulation caused a significant increase in theta (*p* = 0.003), alpha (*p* = 0.018) and beta bands (*p* = 0.008), respectively compared with the sham group (Figure 6A and Table 1A, 1B). All other oscillatory bands showed non-significant changes (*p* > 0.025) in spontaneous absolute power across all stimulation groups after TES compared to sham group (Figure 6A and Table 1A). In the anesthetized state, repetitive prolonged TES yielded non-significant changes (*p* > 0.025) in spontaneous absolute power (Figure 6B and Table 1B) across all oscillatory bands and stimulation groups compared to the sham group.

**TABLE 1A:** Contra primary visual cortex (Vcx) *p*-values from comparison of post-stimulation absolute power between stimulated rd10 and sham rd10 mice in awake state.

Groups	*P*-values							
	
	Delta	Theta	Alpha	Beta	Gamma 1	Gamma 2	Gamma 3	Gamma 4
2 Hz-Sham	0.294	0.484	0.4	0.398	0.538	0.548	0.326	0.198
10 Hz-Sham	0.07	0.003*	0.018*	0.008*	0.182	0.298	0.162	0.12
20 Hz-Sham	0.23	0.191	0.243	0.237	0.43	0.06	0.04	0.028

**P-values with asterisk are significant while p-values without asterisk are non-significant.**

**TABLE 1B:** Contra primary visual cortex (Vcx) *p*-values from comparison of post-stimulation absolute power between stimulated rd10 and sham rd10 mice in anesthetized state.

Groups	*P*-Values							
	
	Delta	Theta	Alpha	Beta	Gamma 1	Gamma 2	Gamma 3	Gamma 4
2 Hz-Sham	0.509	0.708	0.894	0.325	0.486	0.245	0.271	0.3
10 Hz-Sham	0.414	0.117	0.064	0.044	0.562	0.163	0.292	0.22
20 Hz-Sham	0.23	0.308	0.261	0.247	0.382	0.233	0.404	0.789

**P-values without asterisk are non-significant.**

**FIGURE 7:**
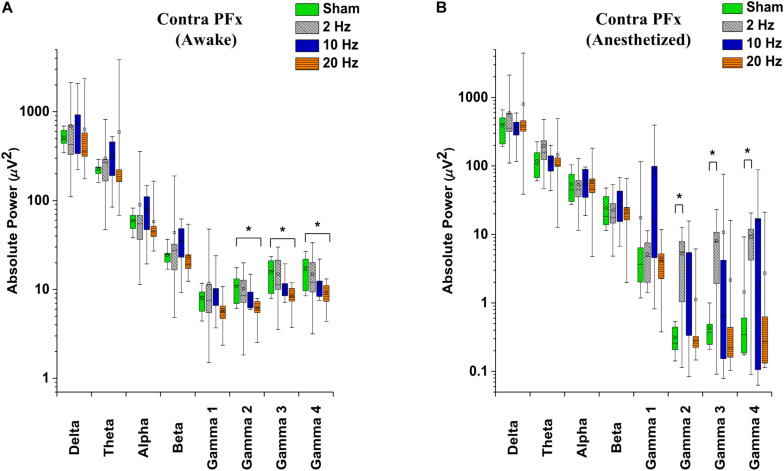
Box-plots showing post-stimulation changes in spontaneous absolute power in the pre-frontal cortex of rd10 mice during awake **(A)** and anesthetized **(B)** brain states. Absolute power is represented in a log scale. All results were compared to the sham group. All *p*-values were adjusted by Holm–Bonferroni method. * denotes significant change in spontaneous absolute power compared to sham group. Short horizontal lines embedded in each boxplot represent the median while the small square symbols represent the mean spontaneous absolute power of each group. Sham (*n* = 8), 2 Hz (*n* = 8), 10 Hz (*n* = 8), 20 Hz (*n* = 9).

In contra PFx of awake rd10 mice, significant decrease in spontaneous absolute power was observed after 20 Hz stimulation specifically in medium gamma (gamma 2; *p* = 0.006), high gamma (gamma 3; *p* = 0.007), and ultra-high gamma (gamma 4; *p* = 0.008) compared to the sham group (Figure 7A and Table 2A). On investigating spontaneous absolute power in contra PFx during the anesthetized brain state, only 2 Hz stimulation caused significant increase (*p* > 0.025) in spontaneous absolute power of medium gamma (gamma 2; *p* = 0.007), high gamma (gamma3; *p* = 0.013), and ultra-high gamma (gamma 4; *p* = 0.009) bands, respectively compared to the sham group (Figure 7B and Table 2B).

**TABLE 2A:** Contralateral pre-frontal cortex (Contra PFx) *p*-values from comparison of post-stimulation absolute power between stimulated rd10 and sham rd10 mice in awake state.

Groups	*P*-Values							
	
	Delta	Theta	Alpha	Beta	Gamma 1	Gamma 2	Gamma 3	Gamma 4
2 Hz-Sham	0.452	0.381	0.431	0.349	0.46	0.823	0.872	0.626
10 Hz-Sham	0.332	0.139	0.211	0.121	0.423	0.168	0.083	0.09
20 Hz-Sham	0.632	0.408	0.944	0.816	0.105	0.006*	0.007*	0.008*

**P-values with asterisk are significant while p-values without asterisk are non-significant.**

**TABLE 2B:** Contralateral pre-frontal cortex (Contra PFx) *p*-values from comparison of post-stimulation absolute power between stimulated rd10 and sham rd10 mice in anesthetized state.

Groups	*P*-Values							
	
	Delta	Theta	Alpha	Beta	Gamma 1	Gamma 2	Gamma 3	Gamma 4
2 Hz-Sham	0.389	0.16	0.951	0.785	0.391	0.007*	0.013*	0.009*
10 Hz-Sham	0.636	0.902	0.767	0.502	0.237	0.127	0.288	0.205
20 Hz-Sham	0.417	0.614	0.807	0.901	0.333	0.271	0.366	0.643

**P-values with asterisk are significant while p-values without asterisk are non-significant.**

### Effects of Repetitive Prolonged TES on Spontaneous Coherence in Rd10 Mice

**FIGURE 8:**
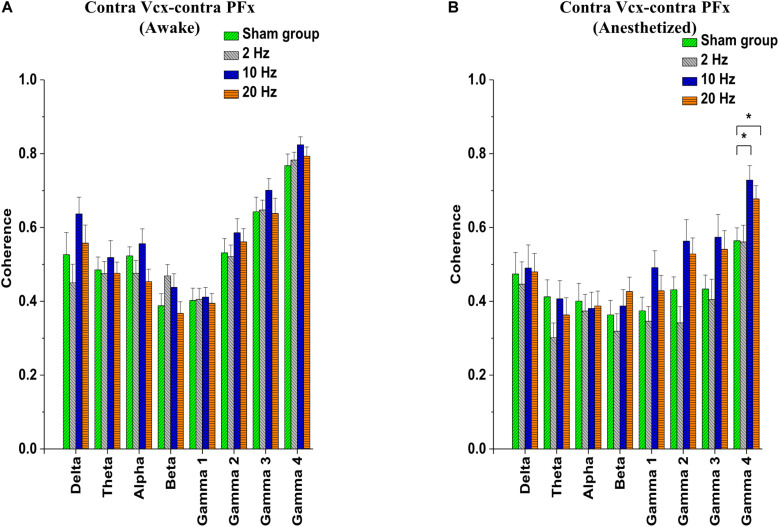
Bar-plots showing post-stimulation changes in contralateral primary visual cortex (contra Vcx)- contralateral pre-frontal cortex (contra PFx) spontaneous coherence of rd10 mice during awake **(A)** and anesthetized **(B)** brain states. Spontaneous coherence is represented as mean ± standard error of the mean (SEM). All results were compared to the sham group. All *p*-values were adjusted by Holm–Bonferroni method. * denotes significant change in spontaneous coherence compared to sham group. Sham (*n* = 8), 2 Hz (*n* = 8), 10 Hz (*n* = 8), 20 Hz (*n* = 9).

Electrocorticogram signals were used to analyze changes in spontaneous coherence after 5 days of prolonged TES in awake and anesthetized rd10 mice. Of interest to us in the present study was the coherence in neural activity between the contra Vcx and the contra PFx. It was revealed that prolonged TES caused no significant change (*p* > 0.025) in contra Vcx-contra PFx coherence in the awake brain state of rd10 mice across all frequency bands and stimulated groups compared to sham group (Figure 8A and Table 3A). However, in the anesthetized state only 10 and 20 Hz stimulation caused a significant increase (*p* < 0.025) in the mean spontaneous coherence compared to the sham group (Figure 8B and Table 3B). For 10 Hz stimulation group, there was a significant increase in mean spontaneous coherence of ultra-high gamma band (gamma 4; *p* = 0.003; Figure 8B and Table 3B). Similarly, 20 Hz stimulation frequency also caused a significant increase (*p* < 0.025) in mean coherence in only ultra-high gamma (gamma 4; *p* = 0.02) oscillations compared with sham (Figure 8B and Table 3B). 2 Hz stimulation frequency produced no significant change (*p* > 0.025) in contra Vcx-contra PFx spontaneous coherence across all analyzed oscillatory bands in both awake and anesthetized states compared with sham group (Figure 8A and Table 3B).

**TABLE 3A:** *P*-values from comparison of post-stimulation contralateral primary visual cortex (contra Vcx) – contralateral pre-frontal cortex (contra PFx) coherence between stimulated rd10 and sham rd10 mice in awake state.

Groups	*P*-Values							
	
	Delta	Theta	Alpha	Beta	Gamma 1	Gamma 2	Gamma 3	Gamma 4
2 Hz-Sham	0.343	0.831	0.281	0.079	0.947	0.836	0.925	0.693
10 Hz-Sham	0.155	0.566	0.488	0.313	0.833	0.327	0.262	0.153
20 Hz-Sham	0.687	0.84	0.11	0.652	0.861	0.576	0.933	0.524

**P-values without asterisk are non-significant.**

**TABLE 3B:** *P*-values from comparison of post-stimulation contralateral primary visual cortex (contra Vcx) – contralateral pre-frontal cortex (contra PFx) coherence between stimulated rd10 and sham rd10 mice in anesthetized state.

Groups					*P*-Values			

	Delta	Theta	Alpha	Beta	Gamma 1	Gamma 2	Gamma 3	Gamma 4
2 Hz-Sham	0.745	0.079	0.685	0.477	0.61	0.117	0.667	0.953
10 Hz-Sham	0.853	0.934	0.762	0.691	0.055	0.06	0.06	0.003*
20 Hz-Sham	0.941	0.459	0.836	0.267	0.342	0.09	0.097	0.02*

**P-values with asterisk are significant while p-values without asterisk are non-significant.**

## Discussion

### Experimental Design With Sham Control

We employed the use of sham rd10 controls in our experimental design because we wanted to evaluate the effect of our TES procedure in an unbiased fashion by ensuring that the observed modulatory effects are due to the applied electrical stimulation instead of being caused by other ancillary factors. Sham controls are documented to be widely used in basic and clinical research (Naycheva et al., 2013; Bola et al., 2014; Zheng et al., 2017). Moreover, a good number of studies in retinal electrical stimulation genre including preclinical studies on rodents and clinical studies have also employed the use of sham controls (Henrich-Noack et al., 2013; Sehic et al., 2016; Bittner et al., 2017; Schatz et al., 2017). Furthermore, we have used only rd10 mice as opposed to the wild type C57BL/6 mice because we wanted to ensure uniformity in the mice genotype for comparison of our ECoG results and to avoid unexpected ECoG results with wild type mice which for instance could possibly arise from genotype, or asymptomatic eye damage during animal housing or from other circumstances which could go unnoticed thus leading to false negative ECoG results. Consequently, to avoid such incidents we thought it wise to ensure that all animals used in our present study had retinal degeneration.

### Pre-stimulation Groups as Second Control

In the absence of wild type C57BL/6 mice we used the pre-stimulation ECoG data of the sham and stimulated animals, respectively as a second control to assess the modulatory effects of TES on the central nerves system. We compared the pre-stimulation ECoG signal of each stimulation group (2, 10, and 20 Hz) with the pre-stimulation ECoG signal of the sham group in both contra Vcx and contra PFx. For both awake anesthetized states of contra Vcx, there was no significant difference (*p* > 0.05) in pre-stimulation ECoG power across all stimulation groups compared with sham (Supplementary Figure S1). Similarly, for both awake and anesthetized states in contra PFx, there was no significant difference (*p* > 0.05) in pre-stimulation ECoG power across all stimulation groups compared with sham (Supplementary Figure S2). Coherence analysis also showed that for both awake and anesthetized states, there was no significant difference (*p* > 0.05) in contra Vcx-contra PFx pre-stimulation coherence between each stimulation groups and sham group (Supplementary Figure S3). The non-significant difference between the pre-stimulation ECoG data of sham rd10 and stimulated rd10 (Supplementary Figures S1, S3) also serves as evidence to demonstrate that the retinal function was the same for all mice before TES because it has been previously shown that the Vcx response is of retinal origin (Foik et al., 2015). Thus, the retinal function correlates with the response from the visual cortex. Moreover, at the start of our experiment all rd10 mice used were aged P60–P90. At this age all photoreceptors and their functions in rd10 are lost in fact it has been shown that retinal degeneration in rd10 starts as early as P18 (Gargini et al., 2007).

### Transcorneal Electrical Stimulation

Transcorneal electrical stimulation is a technique that has been reported to treat various ophthalmological diseases in humans such as Stargardt’s disease, Best dystrophy, and various optic neuropathies (Fujikado et al., 2006; Ozeki et al., 2013). TES has been proven to activate primary visual cortex with structural and functional improvements in primary visual cortex of humans (Timothy and Morley, 2008) and rodent models (Xie et al., 2012). The present study focused on the effect of a prolonged electrical stimulation paradigm on Vcx and PFx. Specifically, we were interested in going beyond the previous findings of other researchers in the field which focused mostly on the effect of electrical stimulation on retinal neurons. Therefore, the goal of the present study was to demonstrate that the effect of TES is not limited to retina neurons, but goes beyond the eye and extends to both the visual (contra Vcx) and non-visual centers (contra PFx) of the brain. In this regard, we studied electrophysiology parameters not only in the awake state but also in the anesthetized state, thus providing a basis for comparing the brain’s response to TES at different states. Currently there are very few available chronic systems of retinal implants (i.e., epiretinal, subretinal, suprachoroidal) in animals, or almost none. Thus, to achieve chronic electrical stimulation paradigm in the present study, the only possible setup was to make use of the non-invasive nature of TES (Morimoto et al., 2012; Ma et al., 2014; Sergeeva et al., 2015).

### ECoG Response From TES-Triggered Excitation of PFx and Vcx Is Dependent on Stimulation Frequency and Brain State

The present study investigated post-stimulation ECoG responses in the pre-frontal cortex and primary visual cortex of awake and anesthetized rd10 mice stimulated by TES. Analysis was based on alterations in spontaneous absolute power and spontaneous coherence of oscillatory bands under varying stimulation frequencies. Firstly, the results reported here demonstrate that prolonged TES is able to excite the pre-frontal and primary visual cortex of rd mice. Secondly prolonged TES modulates cortical properties of power and coherence in brain oscillations of rd mice depending on the frequency used to stimulate the mice retina. Thirdly, the effect of prolonged TES was different depending on the brain state (that is; in awake and anesthetized brain states). Following 5 days prolonged TES in awake rd mice, 10 Hz stimulation showed a more profound effect in exciting the primary visual cortex as evident by the increase in spontaneous absolute power of theta, alpha, and beta band oscillations, respectively compared to sham rd10 mice. Alpha oscillations are known to be the most predominant oscillatory rhythm during resting state (Weisz et al., 2011). Increased spontaneous firing rates in neurons of the CNS have been noted to be a unique feature in disease conditions especially in neurodegenerative diseases. Specifically, it has been reported that the neurons of the primary visual cortex in retinal degeneration model S334ter rats fired more frequently compared to normal rats (Wang et al., 2016). Furthermore, it has been hypothesized that increased spontaneous firing in central auditory neurons is a key factor for the development of tinnitus (Mielczarek et al., 2016). Increased power of alpha oscillations has also been reported to activate the brain actively inhibiting interfering or irrelevant processes in the brain (Fresnoza et al., 2018). From the present study it can be suggested that the observed increased absolute power of alpha oscillations in the primary visual cortex after 10 Hz prolonged TES, possibly suppresses the maladaptive spontaneous firing previously reported in rd model. From previous study alterations of beta oscillatory activity in non-somatomotor areas (frontal, parietal, and visual) have been associated with visual perception (Donner et al., 2007). In the present study, the observed increase in beta oscillations after 10 Hz stimulation in the primary visual cortex of awake rd10 mice could possibly serve to index changes in the neural activity of stimulated rd mice occurring after prolonged TES compared to sham stimulated rd10 mice. Theta oscillations have been shown to be important for working memory and information coding (Benchenane et al., 2010). Our present study reported increased theta power in the primary visual cortex of rd10 mice which could play a role in encoding visual information sent to the brain for interpretation after 10 Hz prolonged TES. Furthermore, this increased theta activity could have a memory-based function and strengthen neuroplastic interactions in population neurons. Possibly in the anesthetized brain state, the primary visual cortex of rd10 mice was not sufficiently activated by prolonged TES thus no significant changes in absolute power was observed in all stimulation groups.

Meanwhile, in the pre-frontal cortex of awake rd10 mice, the present study showed that high stimulation frequency of 20 Hz significantly decreases spontaneous absolute power of medium-, high- and ultra-high gamma oscillations, respectively compared to sham rd10 mice. On the contrary in the same region (pre-frontal cortex) of anesthetized animals, very low stimulation frequency of 2 Hz was shown to be sufficient to significantly increase the spontaneous absolute power of the same medium-, high- and ultra-high gamma oscillations, respectively. At this juncture, it is important to state that the prefrontal cortex coordinates multiple functional networks in the brain for instance, circuits that connect the pre-frontal cortex to the primary visual cortex in order to promote higher cognitive functions including learning, problem-solving and reasoning. Thus, in PFx, the observed increase in spontaneous absolute power of the same medium-, high-, and ultra-high gamma oscillations, respectively in anesthetized rd10 mice after 2 Hz prolonged TES could possibly impact the rd brain with improved cognitive abilities. Although, this may be verified by further investigations such as behavioral tests on stimulated rd10 mice compared with sham rd10 mice.

Gamma oscillations in the range > 25 Hz have been linked to altered states of consciousness and rapid eye movement sleep (Beauregard and Paquette, 2008). Increased gamma activity has also been suggested to play lead roles in binding and unifying multiple inputs from a diverse area (Engel and Singer, 2001). This was reflected in the present study where we observed that anesthetized rd10 mice displayed increased spontaneous coherence between the primary visual cortex and the pre-frontal cortex in ultra-high gamma oscillations after 10 and 20 Hz prolonged TES, respectively compared to sham group. To further corroborate our findings, it has also been reported that high levels of inter-regional coherence in gamma oscillations indicate a highly aroused brain (Engel and Singer, 2001). Thus, from the present study we suggest that the observed increase in coherence between the primary visual cortex and the pre-frontal cortex (after 10 and 20 Hz stimulation) might be reflective of increased synchronization and prolonged TES could impact the rd10 mice brain with the capacity of enhanced neural information processing.

The differences in the effect of prolonged TES in awake and anesthetized states could possibly be explained in terms of variations in cortical processing between the two brain states. These differences could be the result of the anesthesia influence which potentially reduced the brain responsiveness to prolonged TES particularly in the primary visual cortex. These differences have been previously reported in the visual cortex of awake and anesthetized rodents (Keller et al., 2012). Moreover, it has been established that anesthesia has profound effects on neurons for example, glial cell activities and neural activity have been observed to be suppressed and these effects have also been extended to large-scale neural networks (Durand et al., 2016).

### Implications for Retinal Prostheses

In summary, the results reported in the present study suggest that repetitive prolonged TES is able to modulate the resting state brain activity of rd mice in a frequency dependent manner and the consequent power and coherence changes in neural oscillations are linked to the brain state as well as the excited brain region. Following stimulation of the retina, the focus from the existing literatures is predominantly on excitation of the primary visual cortex essentially because the expected goal of retinal stimulation is to provide partial or complete vision restoration in retinal degeneration patients. In our present study we refer to the observed excitation of the primary visual cortex as local cortical excitation while the excitation of the prefrontal cortex we call global cortical excitation. Previous research studies using stimulation techniques such as rTMS (Chung et al., 2016) and tDCS (Sczesny-Kaiser et al., 2016) have demonstrated that brain stimulation (in the motor cortex) can modulate cortical circuits and the terms local and global excitation has also been mentioned (Romero Lauro et al., 2014) in this regard. For our present study both local and global cortical excitability indicate that the effects of retinal stimulation are diffuse, rather than being restricted to the target area, i.e., primary visual cortex. Our study is the first detailed investigation that examines the effect on retinal degeneration brain state under prolonged retinal electrical stimulation. Moreover, our study serves as a starting point for investigating the sustained effect of electrical stimulation of the retina to brain activities. To this end, our first investigation of neural oscillations in prefrontal cortex and primary visual cortex induced by retinal stimulation hopes to inform the research community about the existence of local and global cortical excitation following retinal stimulation. This study suggests that cortical excitability from retinal stimulation should not be undermined and may need to be taken into account whether the retinal stimulation will have an effect on cognitive processing. However, whether this effect will last for a much longer period beyond what was examined in the present study remains to be explored.

## Data Availability Statement

The raw data supporting the conclusions of this article will be made available by the authors, without undue reservation.

## Ethics Statement

All procedures were reviewed and approved by the Animal Research Ethics Sub-Committee at City University of Hong Kong and were carried out in compliance with the Animals (Control of Experiments) Ordinance at Department of Health, Hong Kong Special Administrative Region.

## Author Contributions

SA and LC designed the study and wrote the manuscript. SA and XL performed the *in vivo* experiments and analyzed data. All authors contributed to the article and approved the submitted version.

## Conflict of Interest

The authors declare that the research was conducted in the absence of any commercial or financial relationships that could be construed as a potential conflict of interest.

## Supplementary Material

Supplementary Figure 1 Box-plots showing absolute power in contralateral primary visual cortex (contra Vcx) before prolonged stimulation (pre-stimulation) of rd10 mice. **(A)** Shows the awake state and **(B)** shows the anesthetized state. In both states, there was no significant difference (*p* > 0.05) in pre-stimulation absolute power (*p* > 0.05) in contra Vcx between all experimental groups (2, 10, and 20 Hz) and sham control groups. The statistical significance between all experimental groups and sham control group was tested by one-way ANOVA.

Supplementary Figure 2 Box-plots showing absolute power in contralateral pre-frontal cortex (contra PFx) before prolonged stimulation (pre-stimulation) of rd10 mice. **(A)** Shows the awake state and panel **(B)** shows the anesthetized state. In both states, there was no significant difference (*p* > 0.05) in pre-stimulation absolute power (*p* > 0.05) in contra PFx between all experimental groups (2, 10, and 20 Hz) and sham control groups. The statistical significance between all experimental groups and sham control group was tested by one-way ANOVA.

Supplementary Figure 3 Box-plots showing contralateral primary visual cortex (contra Vcx) – contralateral pre-frontal cortex (contra PFx) coherence before prolonged stimulation (pre-stimulation) of rd10 mice. **(A)** Shows the awake state and **(B)** shows the anesthetized state. In both states, there was no significant difference (*p* > 0.05) in pre-stimulation contra Vcx – contra PFx coherence (*p* > 0.05) between all experimental groups (2, 10, and 20 Hz) and sham control groups. The statistical significance between all experimental groups and sham control group was tested by one-way ANOVA.
