# Supplementary figures and images for: Excitation of the Pre-frontal and Primary Visual Cortex in Response to Transcorneal Electrical Stimulation in Retinal Degeneration Mice
Source: Front Neurosci. 2020 Oct 9;14:572299. doi: 10.3389/fnins.2020.572299 (PMC7584448; doi:10.3389/fnins.2020.572299)

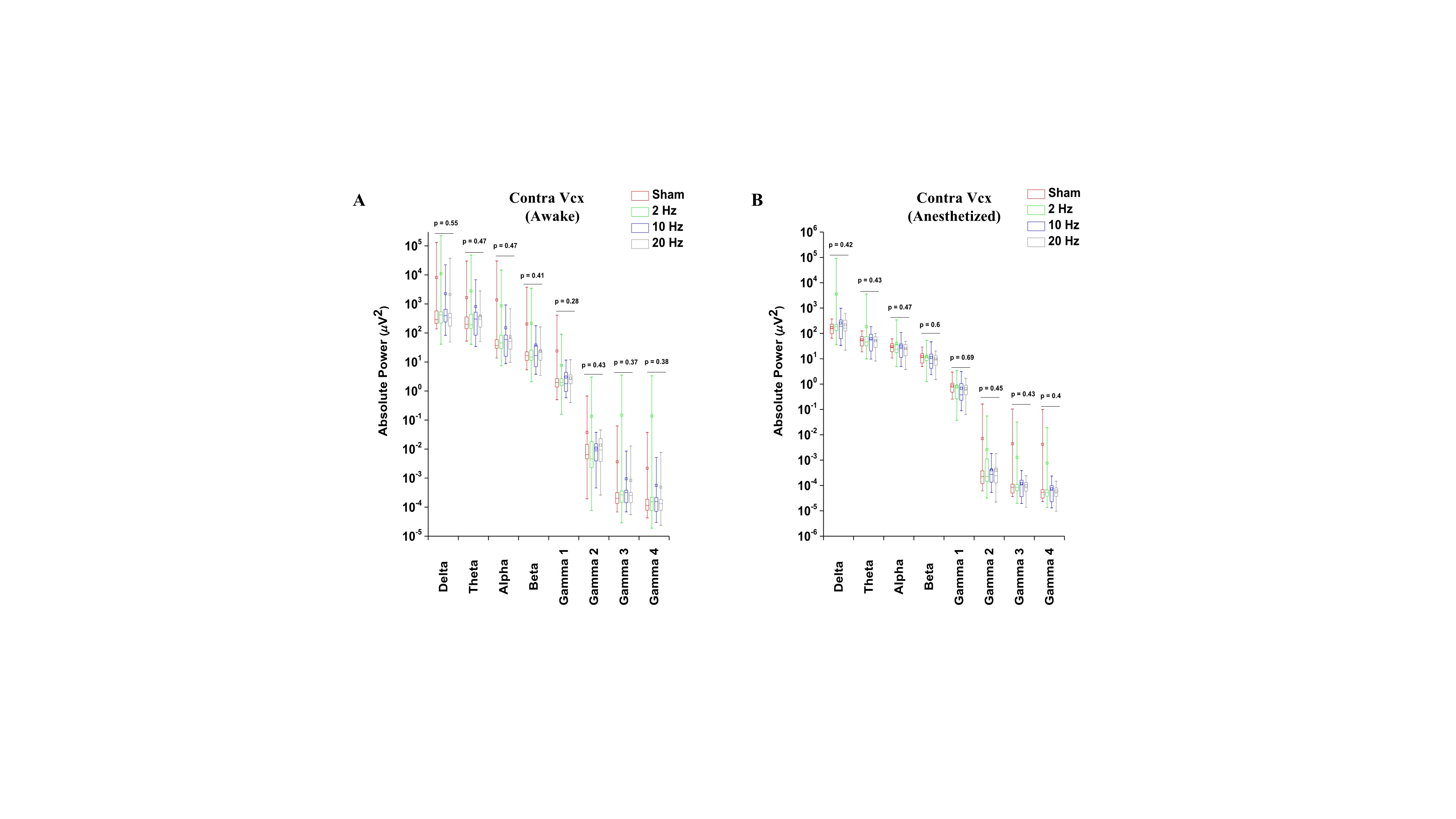

Supplement: Supplementary Figure 1 — Box-plots showing absolute power in contralateral primary visual cortex (contra Vcx) before prolonged stimulation (pre-stimulation) of rd10 mice. (A) Shows the awake state and (B) shows the anesthetized state. In both states, there was no significant difference (p > 0.05) in pre-stimulation absolute power (p > 0.05) in contra Vcx between all experimental groups (2, 10, and 20 Hz) and sham control groups. The statistical significance between all experimental groups and sham control group was tested by one-way ANOVA. [file Image_1.TIF]

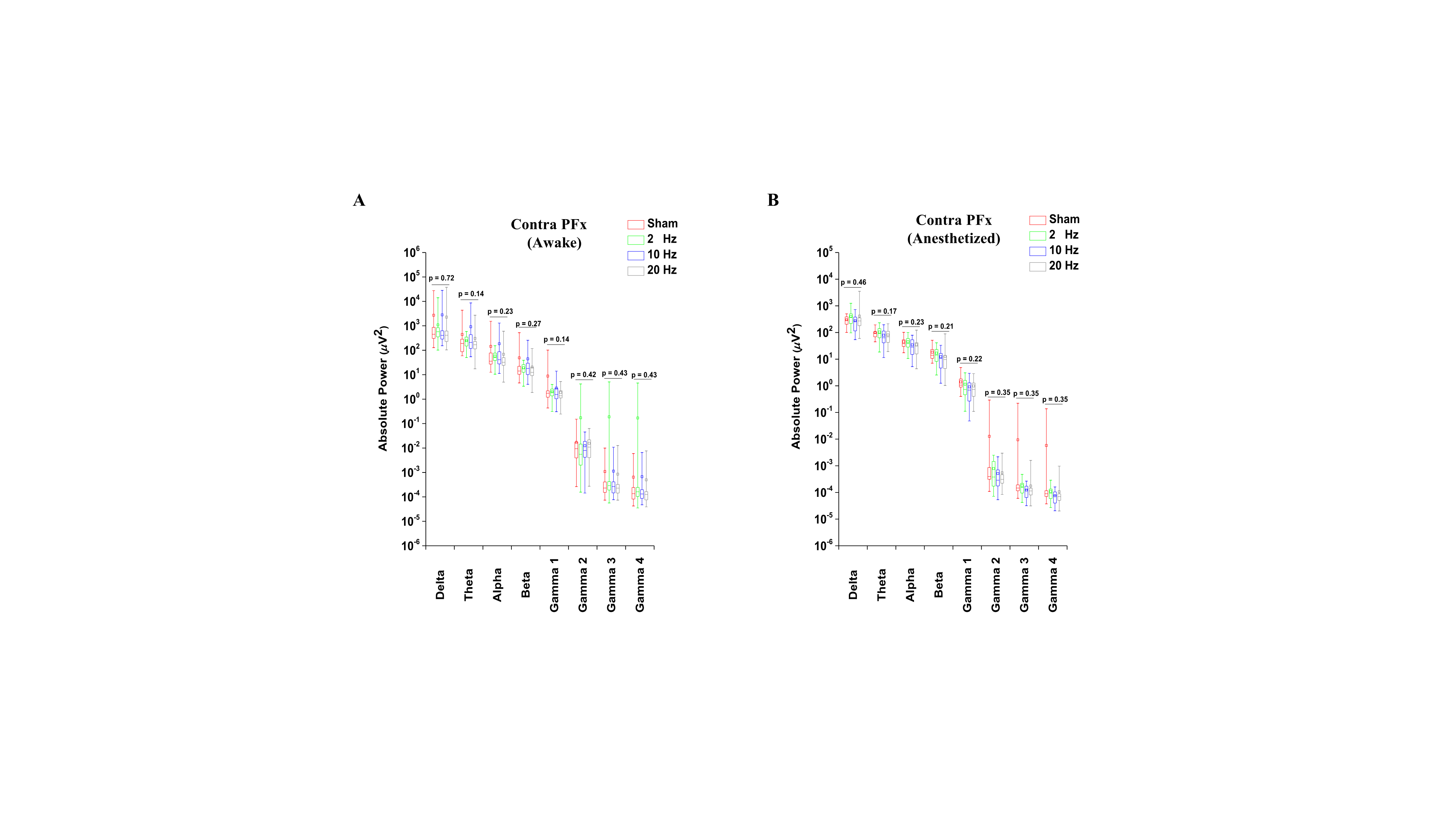

Supplement: Supplementary Figure 2 — Box-plots showing absolute power in contralateral pre-frontal cortex (contra PFx) before prolonged stimulation (pre-stimulation) of rd10 mice. (A) Shows the awake state and panel (B) shows the anesthetized state. In both states, there was no significant difference (p > 0.05) in pre-stimulation absolute power (p > 0.05) in contra PFx between all experimental groups (2, 10, and 20 Hz) and sham control groups. The statistical significance between all experimental groups and sham control group was tested by one-way ANOVA. [file Image_2.TIF]

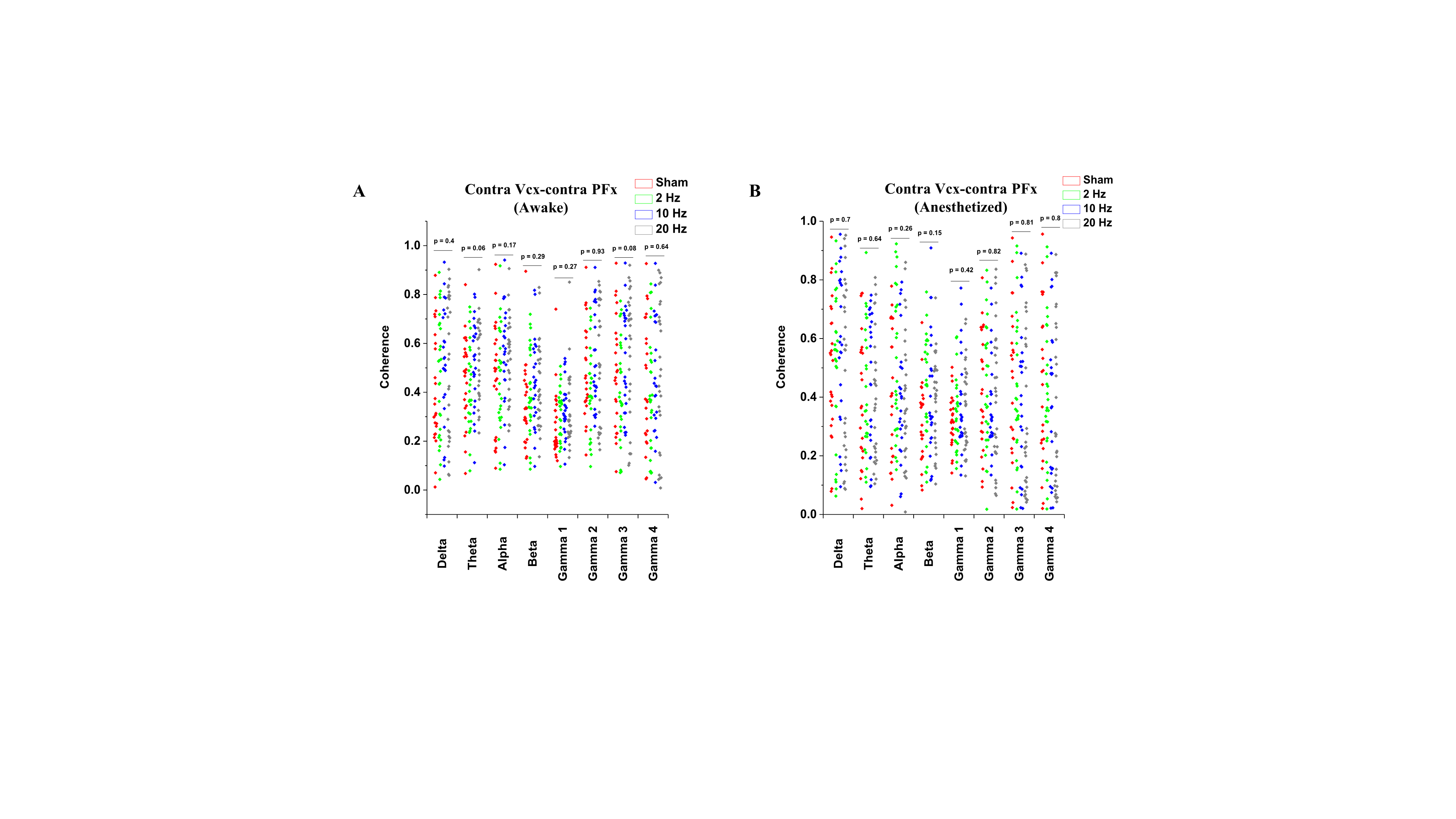

Supplement: Supplementary Figure 3 — Box-plots showing contralateral primary visual cortex (contra Vcx) – contralateral pre-frontal cortex (contra PFx) coherence before prolonged stimulation (pre-stimulation) of rd10 mice. (A) Shows the awake state and (B) shows the anesthetized state. In both states, there was no significant difference (p > 0.05) in pre-stimulation contra Vcx – contra PFx coherence (p > 0.05) between all experimental groups (2, 10, and 20 Hz) and sham control groups. The statistical significance between all experimental groups and sham control group was tested by one-way ANOVA. [file Image_3.TIF]
